# 4-(5-Phenyl-1,2,4-triazolo[3,4-*a*]isoquinolin-3-yl)benzonitrile

**DOI:** 10.1107/S1600536810013012

**Published:** 2010-04-14

**Authors:** F. Nawaz Khan, P. Manivel, K. Prabakaran, Venkatesha R. Hathwar, Mehmet Akkurt

**Affiliations:** a Organic and Medicinal Chemistry Research Laboratory, Organic Chemistry Division, School of Advanced Sciences, VIT University, Vellore 632 014, Tamil Nadu, India; b Solid State and Structural Chemistry Unit, Indian Institute of Science, Bangalore 560 012, Karnataka, India; c Department of Physics, Faculty of Arts and Sciences, Erciyes University, 38039 Kayseri, Turkey

## Abstract

In the title mol­ecule, C_23_H_14_N_4_, the triazoloisoquinoline ring system is nearly planar, with an r.m.s. deviation of 0.038 (2) Å and a maximum deviation of −0.030 (2) Å from the mean plane of the triazole ring C atom which is bonded to the benzene ring. The benzene and phenyl rings are twisted by 57.65 (8) and 53.60 (9)°, respectively, with respect to the mean plane of the triazoloisoquinoline ring system. In the crystal structure, mol­ecules are linked by weak aromatic π–π inter­actions [centroid–centroid distance = 3.8074 (12) Å]. In addition, the crystal structure exhibits a nonclassical inter­molecular C—H⋯N hydrogen bond.

## Related literature

For a related crystal structure, see: Khan *et al.* (2010 ▶).
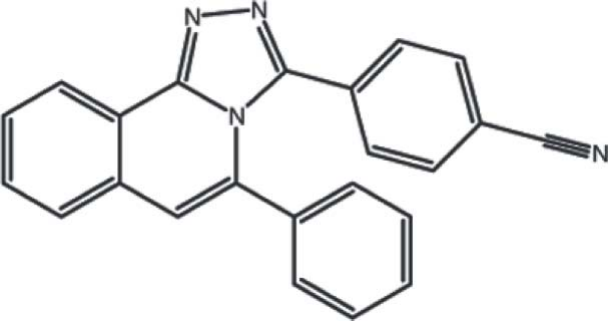


## Experimental

### 

#### Crystal data

C_23_H_14_N_4_
*M* *_r_* = 346.38
Orthorhombic, 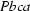

*a* = 7.1614 (3) Å
*b* = 18.0957 (7) Å
*c* = 26.4021 (9) Å
*V* = 3421.5 (2) Å^3^
*Z* = 8
Mo *K*α radiation
μ = 0.08 mm^−1^
*T* = 290 K
0.25 × 0.21 × 0.17 mm

#### Data collection

Oxford Xcalibur Eos (Nova) CCD detector diffractometer
Absorption correction: multi-scan (*CrysAlis PRO RED*; Oxford Diffraction, 2009 ▶) *T*_min_ = 0.959, *T*_max_ = 0.986
14977 measured reflections
3164 independent reflections
1490 reflections with *I* > 2σ(*I*)
*R*_int_ = 0.070

#### Refinement

*R*[*F* ^2^ > 2σ(*F* ^2^)] = 0.046
*wR*(*F* ^2^) = 0.100
*S* = 0.81
3164 reflections
244 parameters
H-atom parameters constrained
Δρ_max_ = 0.15 e Å^−3^
Δρ_min_ = −0.20 e Å^−3^

### 

Data collection: *CrysAlis PRO CCD* (Oxford Diffraction, 2009 ▶); cell refinement: *CrysAlis PRO CCD*; data reduction: *CrysAlis PRO RED* (Oxford Diffraction, 2009 ▶); program(s) used to solve structure: *SHELXS97* (Sheldrick, 2008 ▶); program(s) used to refine structure: *SHELXL97* (Sheldrick, 2008 ▶); molecular graphics: *ORTEP-3* (Farrugia, 1997 ▶); software used to prepare material for publication: *WinGX* (Farrugia, 1999 ▶).

## Supplementary Material

Crystal structure: contains datablocks global, I. DOI: 10.1107/S1600536810013012/rk2199sup1.cif

Structure factors: contains datablocks I. DOI: 10.1107/S1600536810013012/rk2199Isup2.hkl

Additional supplementary materials:  crystallographic information; 3D view; checkCIF report

## Figures and Tables

**Table 1:** Hydrogen-bond geometry (Å, °)

*D*—H⋯*A*	*D*—H	H⋯*A*	*D*⋯*A*	*D*—H⋯*A*
C11—H11⋯N3^i^	0.93	2.50	3.418 (3)	170

Symmetry code: (i) 
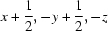
.

**Fig. 1.**
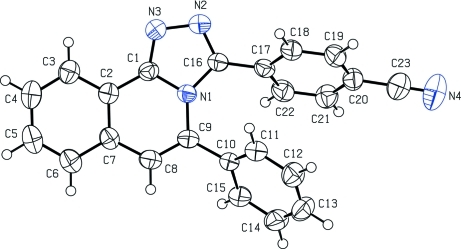
The view of the title molecule with the atom-numbering scheme. Displacement ellipsoids are drawn at the 50% probability level. H atoms are presented as a small spheres of arbitrary radius.

**Fig. 2.**
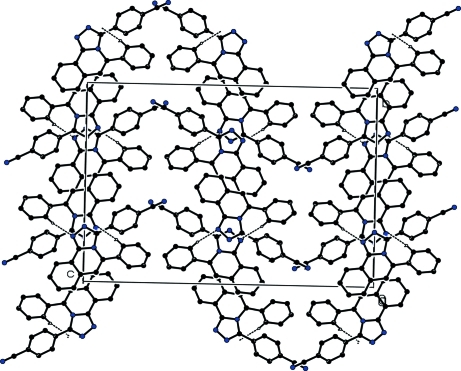
The packing diagram and the hydrogen bonding in the title crystal structure viewed down the [1 0 0] direction. H atoms not involved in the motif shown have been omitted for clarity.

## supplementary crystallographic information

### Comment

As part of our search for new isoquinoline analogues (Khan *et al.*, 2010), we focused on synthesis of titled compounds and the crystal structure is reported.

In the title molecule, Fig. 1, the triazoloisoquinoline ring system (N1-N3/C1-C9/C16) is nearly planar, with an r.m.s. deviation of 0.038 (2)Å and a maximum deviation of -0.030 (2)Å from the mean plane for the triazole ring C16 atom which is bonded to the benzene ring (C17-C22). The benzene (C17-C22) and phenyl (C10-C15) rings are twisted by 57.65 (8)° and 53.60 (9)°, respectively, with respect to the mean plane of the triazoloisoquinoline ring system. The benzene (C17-C22) and phenyl (C10-C15) rings make a dihedral angle of 29.10 (11)° with each other.

Molecular conformation is stabilized by a weak π–π interaction [Cg4···Cg5 = 3.8229 (14)Å, where are Cg4 and Cg5 are centroids of the C10-C15 and C17-C22 rings, respectively]. In the crystal structure, the molecules are linked by weak aromatic π–π interactions [Cg1···Cg1^ii^ = 3.8074 (12)Å, symmetry code: (ii) *x*-1/2, 1/2-*y*, -*z*. Cg1 is the centroid of the N1-N3/C1/C16 ring]. In addition, the crystal structure exhibits an intermolecular non-classical C–H···N hydrogen bond (Table 1, Fig. 2).

### Experimental

2-(3-Phenylisoquinolin-1-yl)hydrazine (1 mmol) was condensed with 4-formylbenzonitrile (1.1 mmol) under refluxing conditions isopropanol (10 ml) solvent to give the corresponding hydrazone in high yield. After removal of solvent the compound was then oxidatively cyclized in nitrobenzene (10 ml) at 473 K. The product was recrystallized from dichlomethane to give block-shaped crystals.

### Refinement

All H atoms were placed in calculated positions with C–H = 0.93Å and were included in the refinement in the riding model approximation, with *U_iso_*(H) = 1.2*U_eq_*(C).

Pure diffraction experiment (ratio observed/unique reflections 47%) we explain by weak diffraction of the crystal.

### Crystal data

**Table:**

C_23_H_14_N_4_	*F*(000) = 1440
*M**_r_* = 346.38	*D*_x_ = 1.345 Mg m^−^^3^
Orthorhombic, *P**b**c**a*	Mo *K*α radiation, λ = 0.71073 Å
Hall symbol: -P 2ac 2ab	Cell parameters from 1235 reflections
*a* = 7.1614 (3) Å	θ = 1.6–20.4°
*b* = 18.0957 (7) Å	µ = 0.08 mm^−^^1^
*c* = 26.4021 (9) Å	*T* = 290 K
*V* = 3421.5 (2) Å^3^	Block, colourless
*Z* = 8	0.25 × 0.21 × 0.17 mm

### Data collection

**Table:**

Oxford Xcalibur Eos (Nova) CCD detector diffractometer	3164 independent reflections
Radiation source: Enhance (Mo) X-ray Source	1490 reflections with *I* > 2σ(*I*)
graphite	*R*_int_ = 0.070
ω scans	θ_max_ = 25.5°, θ_min_ = 3.1°
Absorption correction: multi-scan (*CrysAlis PRO RED*; Oxford Diffraction, 2009)	*h* = −8→7
*T*_min_ = 0.959, *T*_max_ = 0.986	*k* = −21→21
14977 measured reflections	*l* = −31→31

### Refinement

**Table:**

Refinement on *F*^2^	Primary atom site location: structure-invariant direct methods
Least-squares matrix: full	Secondary atom site location: difference Fourier map
*R*[*F*^2^ > 2σ(*F*^2^)] = 0.046	Hydrogen site location: inferred from neighbouring sites
*wR*(*F*^2^) = 0.100	H-atom parameters constrained
*S* = 0.81	*w* = 1/[σ^2^(*F*_o_^2^) + (0.0413*P*)^2^] where *P* = (*F*_o_^2^ + 2*F*_c_^2^)/3
3164 reflections	(Δ/σ)_max_ < 0.001
244 parameters	Δρ_max_ = 0.15 e Å^−^^3^
0 restraints	Δρ_min_ = −0.20 e Å^−^^3^

### Special details

**Table:**

Geometry. All s.u.'s (except the s.u. in the dihedral angle between two l.s. planes) are estimated using the full covariance matrix. The cell s.u.'s are taken into account individually in the estimation of s.u.'s in distances, angles and torsion angles; correlations between s.u.'s in cell parameters are only used when they are defined by crystal symmetry. An approximate (isotropic) treatment of cell s.u.'s is used for estimating s.u.'s involving l.s. planes.
Refinement. Refinement of *F*^2^ against ALL reflections. The weighted *R*-factor wR and goodness of fit *S* are based on *F*^2^, conventional *R*-factors *R* are based on *F*, with *F* set to zero for negative *F*^2^. The threshold expression of *F*^2^ > σ(*F*^2^) is used only for calculating *R*-factors(gt) etc. and is not relevant to the choice of reflections for refinement. *R*-factors based on *F*^2^ are statistically about twice as large as those based on *F*, and *R*-factors based on ALL data will be even larger.

### Fractional atomic coordinates and isotropic or equivalent isotropic displacement parameters (Å^2^)

**Table:**

	*x*	*y*	*z*	*U*_iso_*/*U*_eq_	
N1	0.6627 (2)	0.33099 (9)	0.03366 (6)	0.0370 (4)	
N2	0.5820 (3)	0.22467 (10)	−0.00072 (7)	0.0510 (5)	
N3	0.6063 (2)	0.27481 (11)	−0.03950 (7)	0.0495 (5)	
N4	0.5390 (3)	0.08980 (15)	0.27259 (9)	0.0927 (9)	
C1	0.6539 (3)	0.33812 (13)	−0.01851 (8)	0.0395 (5)	
C2	0.6915 (3)	0.40722 (12)	−0.04237 (8)	0.0407 (6)	
C3	0.6888 (3)	0.41638 (14)	−0.09495 (8)	0.0490 (6)	
H3	0.6586	0.3766	−0.1157	0.059*	
C4	0.7302 (3)	0.48368 (15)	−0.11611 (9)	0.0570 (7)	
H4	0.7297	0.4894	−0.1511	0.068*	
C5	0.7728 (3)	0.54308 (14)	−0.08507 (9)	0.0581 (7)	
H5	0.8002	0.5887	−0.0996	0.070*	
C6	0.7754 (3)	0.53585 (13)	−0.03328 (9)	0.0526 (6)	
H6	0.8030	0.5764	−0.0130	0.063*	
C7	0.7364 (3)	0.46741 (12)	−0.01110 (8)	0.0416 (6)	
C8	0.7527 (3)	0.45523 (12)	0.04249 (8)	0.0454 (6)	
H8	0.7853	0.4952	0.0628	0.054*	
C9	0.7238 (3)	0.38972 (12)	0.06498 (8)	0.0387 (6)	
C10	0.7671 (3)	0.37673 (11)	0.11909 (8)	0.0374 (5)	
C11	0.8907 (3)	0.32192 (12)	0.13406 (8)	0.0456 (6)	
H11	0.9426	0.2903	0.1101	0.055*	
C12	0.9365 (3)	0.31454 (14)	0.18466 (9)	0.0562 (7)	
H12	1.0180	0.2774	0.1948	0.067*	
C13	0.8625 (4)	0.36161 (15)	0.22007 (9)	0.0645 (8)	
H13	0.8935	0.3561	0.2541	0.077*	
C14	0.7438 (4)	0.41647 (15)	0.20553 (9)	0.0595 (7)	
H14	0.6960	0.4490	0.2295	0.071*	
C15	0.6944 (3)	0.42380 (13)	0.15537 (9)	0.0478 (6)	
H15	0.6114	0.4608	0.1458	0.057*	
C16	0.6137 (3)	0.25837 (12)	0.04252 (8)	0.0406 (6)	
C17	0.5906 (3)	0.22246 (12)	0.09194 (8)	0.0403 (5)	
C18	0.6918 (3)	0.15870 (13)	0.10218 (8)	0.0480 (6)	
H18	0.7701	0.1391	0.0775	0.058*	
C19	0.6771 (3)	0.12414 (12)	0.14857 (9)	0.0523 (6)	
H19	0.7474	0.0821	0.1554	0.063*	
C20	0.5575 (3)	0.15217 (13)	0.18497 (8)	0.0480 (6)	
C21	0.4514 (3)	0.21414 (13)	0.17476 (8)	0.0524 (6)	
H21	0.3693	0.2324	0.1990	0.063*	
C22	0.4678 (3)	0.24882 (13)	0.12834 (8)	0.0503 (6)	
H22	0.3957	0.2903	0.1214	0.060*	
C23	0.5459 (3)	0.11720 (15)	0.23405 (10)	0.0629 (8)	

### Atomic displacement parameters (Å^2^)

**Table:**

	*U*^11^	*U*^22^	*U*^33^	*U*^12^	*U*^13^	*U*^23^
N1	0.0393 (10)	0.0337 (11)	0.0381 (11)	−0.0026 (8)	−0.0031 (8)	−0.0012 (9)
N2	0.0631 (13)	0.0439 (12)	0.0459 (12)	−0.0068 (10)	−0.0062 (10)	−0.0041 (11)
N3	0.0614 (13)	0.0457 (13)	0.0414 (11)	−0.0057 (10)	−0.0054 (9)	0.0003 (10)
N4	0.0964 (19)	0.124 (2)	0.0576 (16)	0.0151 (16)	0.0099 (14)	0.0261 (16)
C1	0.0394 (14)	0.0404 (15)	0.0387 (13)	−0.0003 (11)	−0.0054 (10)	−0.0027 (12)
C2	0.0357 (13)	0.0443 (15)	0.0421 (14)	0.0023 (11)	−0.0010 (10)	0.0016 (12)
C3	0.0463 (15)	0.0544 (17)	0.0463 (15)	0.0062 (13)	0.0002 (11)	0.0032 (13)
C4	0.0515 (17)	0.0687 (19)	0.0507 (15)	0.0085 (14)	0.0028 (12)	0.0160 (15)
C5	0.0464 (16)	0.0584 (19)	0.0695 (19)	−0.0011 (13)	−0.0002 (13)	0.0241 (15)
C6	0.0500 (15)	0.0465 (16)	0.0613 (17)	−0.0003 (12)	−0.0016 (13)	0.0076 (13)
C7	0.0344 (14)	0.0413 (15)	0.0490 (14)	0.0004 (11)	−0.0046 (11)	0.0072 (12)
C8	0.0461 (14)	0.0387 (15)	0.0513 (15)	−0.0011 (11)	−0.0052 (12)	−0.0054 (12)
C9	0.0347 (14)	0.0368 (15)	0.0445 (13)	−0.0002 (10)	−0.0019 (11)	−0.0060 (11)
C10	0.0399 (14)	0.0336 (13)	0.0386 (13)	−0.0029 (11)	−0.0012 (10)	−0.0018 (11)
C11	0.0441 (14)	0.0436 (15)	0.0492 (15)	−0.0011 (12)	−0.0005 (11)	−0.0047 (12)
C12	0.0519 (16)	0.0604 (18)	0.0563 (17)	0.0036 (13)	−0.0129 (13)	0.0072 (15)
C13	0.075 (2)	0.077 (2)	0.0416 (15)	−0.0091 (16)	−0.0111 (14)	0.0000 (15)
C14	0.0721 (18)	0.0580 (18)	0.0483 (16)	−0.0068 (15)	0.0073 (14)	−0.0115 (14)
C15	0.0507 (15)	0.0429 (15)	0.0497 (15)	0.0013 (12)	0.0008 (12)	−0.0051 (13)
C16	0.0436 (14)	0.0361 (14)	0.0423 (13)	−0.0039 (11)	−0.0025 (11)	−0.0031 (12)
C17	0.0400 (13)	0.0351 (14)	0.0458 (14)	−0.0048 (11)	−0.0039 (11)	−0.0015 (11)
C18	0.0586 (15)	0.0392 (15)	0.0463 (15)	0.0034 (13)	0.0039 (12)	−0.0031 (12)
C19	0.0637 (17)	0.0400 (15)	0.0532 (16)	0.0085 (12)	0.0004 (13)	0.0023 (13)
C20	0.0498 (15)	0.0497 (16)	0.0446 (14)	−0.0053 (13)	−0.0014 (12)	0.0051 (13)
C21	0.0516 (16)	0.0559 (17)	0.0497 (16)	0.0024 (14)	0.0067 (12)	−0.0002 (13)
C22	0.0516 (16)	0.0417 (15)	0.0575 (16)	0.0052 (12)	−0.0014 (13)	0.0000 (13)
C23	0.0591 (18)	0.075 (2)	0.0546 (18)	0.0052 (14)	0.0030 (14)	0.0043 (16)

### Geometric parameters (Å, °)

**Table:**

N1—C16	1.380 (2)	C10—C11	1.387 (3)
N1—C1	1.385 (2)	C11—C12	1.382 (3)
N1—C9	1.416 (2)	C11—H11	0.9300
N2—C16	1.314 (2)	C12—C13	1.371 (3)
N2—N3	1.379 (2)	C12—H12	0.9300
N3—C1	1.318 (3)	C13—C14	1.362 (3)
N4—C23	1.133 (3)	C13—H13	0.9300
C1—C2	1.426 (3)	C14—C15	1.377 (3)
C2—C3	1.398 (3)	C14—H14	0.9300
C2—C7	1.404 (3)	C15—H15	0.9300
C3—C4	1.372 (3)	C16—C17	1.467 (3)
C3—H3	0.9300	C17—C22	1.387 (3)
C4—C5	1.386 (3)	C17—C18	1.389 (3)
C4—H4	0.9300	C18—C19	1.379 (3)
C5—C6	1.374 (3)	C18—H18	0.9300
C5—H5	0.9300	C19—C20	1.384 (3)
C6—C7	1.398 (3)	C19—H19	0.9300
C6—H6	0.9300	C20—C21	1.381 (3)
C7—C8	1.437 (3)	C20—C23	1.445 (3)
C8—C9	1.342 (3)	C21—C22	1.382 (3)
C8—H8	0.9300	C21—H21	0.9300
C9—C10	1.481 (3)	C22—H22	0.9300
C10—C15	1.384 (3)		
			
C16—N1—C1	104.23 (17)	C12—C11—H11	120.1
C16—N1—C9	133.97 (18)	C10—C11—H11	120.1
C1—N1—C9	121.67 (19)	C13—C12—C11	120.5 (2)
C16—N2—N3	108.54 (17)	C13—C12—H12	119.7
C1—N3—N2	107.01 (17)	C11—C12—H12	119.7
N3—C1—N1	110.4 (2)	C14—C13—C12	120.1 (2)
N3—C1—C2	128.7 (2)	C14—C13—H13	119.9
N1—C1—C2	120.8 (2)	C12—C13—H13	119.9
C3—C2—C7	119.7 (2)	C13—C14—C15	120.1 (2)
C3—C2—C1	122.7 (2)	C13—C14—H14	120.0
C7—C2—C1	117.6 (2)	C15—C14—H14	120.0
C4—C3—C2	120.4 (2)	C14—C15—C10	120.7 (2)
C4—C3—H3	119.8	C14—C15—H15	119.7
C2—C3—H3	119.8	C10—C15—H15	119.7
C3—C4—C5	119.7 (2)	N2—C16—N1	109.78 (18)
C3—C4—H4	120.2	N2—C16—C17	123.23 (19)
C5—C4—H4	120.2	N1—C16—C17	126.94 (19)
C6—C5—C4	121.2 (2)	C22—C17—C18	118.8 (2)
C6—C5—H5	119.4	C22—C17—C16	122.4 (2)
C4—C5—H5	119.4	C18—C17—C16	118.8 (2)
C5—C6—C7	119.9 (2)	C19—C18—C17	120.6 (2)
C5—C6—H6	120.0	C19—C18—H18	119.7
C7—C6—H6	120.0	C17—C18—H18	119.7
C6—C7—C2	119.1 (2)	C18—C19—C20	119.9 (2)
C6—C7—C8	122.1 (2)	C18—C19—H19	120.1
C2—C7—C8	118.6 (2)	C20—C19—H19	120.1
C9—C8—C7	124.0 (2)	C21—C20—C19	120.2 (2)
C9—C8—H8	118.0	C21—C20—C23	119.9 (2)
C7—C8—H8	118.0	C19—C20—C23	119.9 (2)
C8—C9—N1	116.90 (19)	C20—C21—C22	119.7 (2)
C8—C9—C10	122.32 (19)	C20—C21—H21	120.2
N1—C9—C10	120.61 (19)	C22—C21—H21	120.2
C15—C10—C11	118.9 (2)	C21—C22—C17	120.8 (2)
C15—C10—C9	119.4 (2)	C21—C22—H22	119.6
C11—C10—C9	121.49 (19)	C17—C22—H22	119.6
C12—C11—C10	119.7 (2)	N4—C23—C20	179.2 (3)
			
C16—N2—N3—C1	0.5 (2)	N1—C9—C10—C15	−131.8 (2)
N2—N3—C1—N1	0.3 (2)	C8—C9—C10—C11	−121.8 (2)
N2—N3—C1—C2	−178.9 (2)	N1—C9—C10—C11	53.2 (3)
C16—N1—C1—N3	−0.9 (2)	C15—C10—C11—C12	1.0 (3)
C9—N1—C1—N3	175.46 (16)	C9—C10—C11—C12	176.0 (2)
C16—N1—C1—C2	178.40 (18)	C10—C11—C12—C13	−0.9 (3)
C9—N1—C1—C2	−5.2 (3)	C11—C12—C13—C14	−0.4 (4)
N3—C1—C2—C3	−2.5 (3)	C12—C13—C14—C15	1.4 (4)
N1—C1—C2—C3	178.35 (17)	C13—C14—C15—C10	−1.3 (4)
N3—C1—C2—C7	178.8 (2)	C11—C10—C15—C14	0.0 (3)
N1—C1—C2—C7	−0.4 (3)	C9—C10—C15—C14	−175.0 (2)
C7—C2—C3—C4	0.2 (3)	N3—N2—C16—N1	−1.0 (2)
C1—C2—C3—C4	−178.5 (2)	N3—N2—C16—C17	176.63 (18)
C2—C3—C4—C5	−0.8 (3)	C1—N1—C16—N2	1.2 (2)
C3—C4—C5—C6	0.4 (3)	C9—N1—C16—N2	−174.51 (19)
C4—C5—C6—C7	0.7 (3)	C1—N1—C16—C17	−176.38 (19)
C5—C6—C7—C2	−1.2 (3)	C9—N1—C16—C17	7.9 (4)
C5—C6—C7—C8	174.57 (19)	N2—C16—C17—C22	−120.5 (2)
C3—C2—C7—C6	0.8 (3)	N1—C16—C17—C22	56.7 (3)
C1—C2—C7—C6	179.55 (19)	N2—C16—C17—C18	57.8 (3)
C3—C2—C7—C8	−175.16 (18)	N1—C16—C17—C18	−125.0 (2)
C1—C2—C7—C8	3.6 (3)	C22—C17—C18—C19	−3.0 (3)
C6—C7—C8—C9	−177.2 (2)	C16—C17—C18—C19	178.6 (2)
C2—C7—C8—C9	−1.5 (3)	C17—C18—C19—C20	1.4 (3)
C7—C8—C9—N1	−3.9 (3)	C18—C19—C20—C21	0.7 (3)
C7—C8—C9—C10	171.32 (18)	C18—C19—C20—C23	−178.3 (2)
C16—N1—C9—C8	−177.6 (2)	C19—C20—C21—C22	−1.2 (3)
C1—N1—C9—C8	7.3 (3)	C23—C20—C21—C22	177.8 (2)
C16—N1—C9—C10	7.0 (3)	C20—C21—C22—C17	−0.4 (3)
C1—N1—C9—C10	−168.06 (17)	C18—C17—C22—C21	2.5 (3)
C8—C9—C10—C15	53.1 (3)	C16—C17—C22—C21	−179.2 (2)

### Hydrogen-bond geometry (Å, °)

**Table:**

*D*—H···*A*	*D*—H	H···*A*	*D*···*A*	*D*—H···*A*
C11—H11···N3^i^	0.93	2.50	3.418 (3)	170

Symmetry codes: (i) *x*+1/2, −*y*+1/2, −*z*.
